# Books Received

**Published:** 1869-07-15

**Authors:** 


### BOOKS RECEIVED.

A PRACTICAL MANUAL ON THE TREATMENT OF CLUB FOOT. By Lewis A. Sayre, M.D., Prof., etc. New York: D. Appleton & Co. 1869. Pp. 91. S. C. Griggs & Co., Chicago. $1.25.

We omit the major part of Dr. Sayer's title page, consisting of a formidable inverted pyramid of accumulated honors, to swell which, he includes his membership of various state, county and city societies, as, also, his official position in one of them. Regarding the book as a means of advertising (albeit but little above the various advertising *dodges* of Quackery), this display is consistent, and entirely in keeping with the following two sentences from the first paragraph of his preface:

"Within the past month I have known of three instances where the tendo capchillis has been divided for talipes varus, and in each case by gentlemen of the highest standing in our profession. No censure is here intended, for the gentleman referred to but followed strictly the teachings of our standard authorities."

Lenient and forbearing author! how we admire thy goodness of heart! How fortunate the three "gentlemen of the highest standing" must deem themselves! One would naturally suppose that the author never found it necessary to divide the great tendon, but in the body of the advertisement he informs us that cases do occur when "it is absolutely essential, as a preliminary measure to all other treatment." Rumor hath it, also, that the author is famous as cutter of tendons, faciæ, and *other things.*

Our author further informs us that:

"*The Seat of Talipes* has always, until recently, been supposed to be at the ankle-joint."

That this is but a man of straw set up to be valiantly knocked down by our advertiser, is known to any medical student who has even looked at the pictures in the text books. But it is entirely in keeping with the general run of advertisements of the baser sort, to which the little book now noticed is own cousin.

---

THIRTEENTH ANNUAL REPORT OF THE BOARD OF GUARDIANS OF THE CHICAGO REFORM SCHOOL, to the Common Council of the City of Chicago, for the year ending March 31, 1869. Pp. 50.

---

ANNUAL ANNOUNCEMENT OF DEPARTMENT OF MEDICINE, Willamette University, Salem, Oregon, Session 1869–70. Address H. Carpenter, M.D., Dean.

---

INCIDENTS OF TRAVEL, to the American Medical Association, at New Orleans, May, 1869. By W. K. Bowling, M.D. From June number of Nashville Journal of Medicine and Surgery.

